# Neuroprotective Effects of Gagam-Sipjeondaebo-Tang, a Novel Herbal Formula, against MPTP-Induced Parkinsonian Mice and MPP^+^-Induced Cell Death in SH-SY5Y Cells

**DOI:** 10.1155/2018/2420809

**Published:** 2018-12-04

**Authors:** Jade Heejae Ko, Ju-Hee Lee, Bobin Choi, Ju-Yeon Park, Young-Won Kwon, Songhee Jeon, Sun-Dong Park, Seung-Nam Kim

**Affiliations:** ^1^College of Korean Medicine, Dongguk University, Goyang 10326, Republic of Korea; ^2^Department of Biomedical Sciences, Center for Creative Biomedical Scientists, Chonnam National University, Gwangju 61469, Republic of Korea

## Abstract

Parkinson's disease is a neurodegenerative disease characterized by progressive cell death of dopaminergic neuron and following neurological disorders. Gagam-Sipjeondaebo-Tang (GST) is a novel herbal formula made of twelve medicinal herbs derived from Sipjeondaebo-Tang, which has been broadly used in a traditional herbal medicine. In the present study, we investigated the effects of GST against 1-methyl-4-phenyl-1,2,3,6-tetrahydropyridine (MPTP)-induced motor abnormalities in mice and 1-methyl-4-phenylpyridinium (MPP^+^)-induced neurotoxicity in SH-SY5Y cell. First, we found that GST alleviated motor dysfunction induced by MPTP, and the result showed dopaminergic neurons recovery in substantia nigra. In the cell experiment, pretreatment with GST increased the cell viability and attenuated apoptotic cell death in MPP^+^-treated SH-SY5Y cells. GST also inhibited reactive oxygen species production and restored the mitochondrial membrane potential loss, which were induced by MPP^+^. Furthermore, GST extract significantly activated ERK and Akt, cell survival-related proteins, in SH-SY5Y cells. The effect of GST preventing mitochondrial dysfunction was antagonized by pretreatment of PD98059 and LY294002, selective inhibitors of ERK and Akt, respectively. Taken together, GST alleviated abnormal motor functions and recovered neuronal cell death, mitochondrial dysfunction, possibly via ERK and Akt activation. Therefore, we suggest that GST may be a candidate for the treatment and prevention of Parkinson's disease.

## 1. Introduction

Parkinson's disease (PD) is characterized by extensive cell death of dopaminergic neuron in the substantia nigra and subsequently develops into other neurological disorders [1]. It has been known that familial and sporadic pathological factors induce neuronal deficits by reactive oxygen stress and following mitochondrial dysfunction [2]. Considering how rapidly population of PD grows, there is comparatively less development of conventional methods so that alternative treatments for PD has been drawing attention [3].

Sipjeondaebo-Tang (SDT; Shi-Quan-Da-Bu-Tang in Chinese and Juzen-Taiho-To in Japanese) is broadly used to treat neurological patients in traditional East Asian herbal medicine [4]. It has been shown that SDT has anti-inflammatory effect in LPS-stimulated RAW cells [5], reduction of toxicity, and regulation of immune system in hepatic tumor [6]. It was also revealed that SDT recovered DNA fragmentation and mitochondrial dysfunction in antitumor drug given mice [7]. Gagam-Sipjeondaebo-Tang (GST) is a novel herbal formula of twelve medicinal herbs derived from SDT. GST is modified formula of SDT with two additional herbs which are* Uncaria rhynchophylla* and* Gastrodia elata* Blume. The two herbs were reported to have positive effect on PD [8, 9] and have been used for patients who have motor dysfunctions in East Asia countries. Furthermore,* Uncaria rhynchophylla* extract has shown neuroprotective effect in MPP^+^-induced SH-SY5Y cells and MPTP-induced mice by increasing cell viability and ameliorating behavioral impairment through dopamine and tyrosine hydroxylase (TH) expression elevation [10]. Anti-apoptotic and antioxidative aspects of* Gastrodia elata* Blume in MPP^+^-induced cytotoxicity dopaminergic cells were also suggested [11]. The earlier studies suggest that GST potentially has neuroprotective effects of dopaminergic neuron and its following motor dysfunctions of PD. Therefore, in this study, we investigated effects of GST on PD and its underlying mechanism using 1-methyl-4-phenyl-1,2,3,6-tetrahydropyridine (MPTP)-induced Parkinsonian mice model and 1-methyl-4-phenylpyridinium (MPP^+^)-induced cell death.

## 2. Materials and Methods

### 2.1. Animals

**Figure 1:**
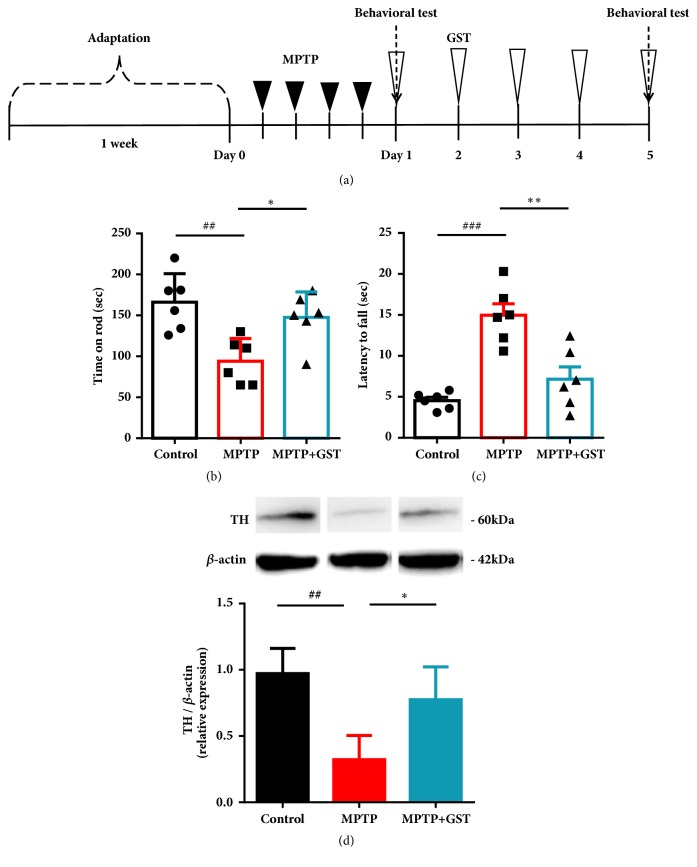
**Effects of GST extract on motor functions in MPTP-induced Parkinsonian mice. **(a) Timeline of the animal test in this study. Filled arrowheads indicate MPTP injections to induce Parkinsonian mice. Blank arrowheads indicate GST treatment. Dashed arrow shows the day of behavioral test. (b) Effects of GST on the duration of MPTP and control mice stayed on the rotating rod. (c) The result of time spent in the pole test. The time spent before mice arrived on the ground from top of the pole was measured. (d) Western blot shows difference of TH level between control group, MPTP group, and GST administered MPTP group. ##* p* < 0.01 and ###* p* < 0.001, compared to control group, *∗ p* < 0.05 and ∗∗* p* < 0.01, compared to MPTP group, analyzed by one-way ANOVA, followed by Bonferroni's* post hoc* test.

Male C57BL/6 mice (Orient-Bio Co., Seongnam, Republic of Korea), 9 weeks of age and weighing 20–25 g each, were used. The animals were housed under a 12/12-h light/dark cycle with free access to food and water. All experiments were approved by the Dongguk University Animal Care Committee for animal welfare (2016-DGU-46) and maintained in strict accordance with guidelines. To develop PD animal model, we used acute MPTP regimen on mice. Briefly, intraperitoneal MPTP injection (20 mg/kg of body weight, Sigma-Aldrich, St. Louis, USA) was performed every 2 hours, four times a day in total. One day after MPTP injection, rotarod test was done and only mice that showed motor function deficits more than 30% compared to control group were divided into experimental groups for this study: control group, MPTP group and MPTP+GST group. 300 mg/kg of GST was given to MPTP+GST group once a day for 5 days by oral administration. There were 6 mice in each group. The same volume of physiological saline was administered to mice in the control group. After the behavioral tests, the mice were sacrificed and the substantia nigra in the brain was removed. Detailed timeline of the animal study is shown in Figure 1(a).

### 2.2. Behavioral Test

We have conducted rotarod test and pole test to examine the motor function of the mice. For the rotarod test, mice were located on a rotating rod accelerating from 5 to 20 rpm for a 240 seconds time period. Each mouse was scored for time they sustained on the rod. The mice that completed the task over 240 seconds scored as 240, which was the maximum value. To examine pole test, mice were located head toward up, close to top of the pole. We recorded the time when the mice's heads completely rotated downward (P1) and when they reached the floor (P2). Falling latency was calculated by subtracting P1 from P2. The maximum time limit was determined as 120 seconds.

### 2.3. Plant Materials and Preparation of GST Extract

The GST was formulated from twelve medicinal herbs:* Panax ginseng* C. A. Meyer,* Atractylodes macrocephala* Koidzumi,* Poria cocos* Wolf,* Glycyrrhiza uralensis* Fischer,* Rehmannia glutinosa* Liboschitz ex Steudel,* Angelica gigas* Nakai,* Cnidium officinale* Makino,* Paeonia lactiflora* Pallas,* Astragalus membranaceus* Bunge,* Bupleurum falcatum* Linne,* Uncaria rhynchophylla*, and* Gastrodia elata* Blume. All herbs were purchased as dried herbs from Omniherb (Seoul, Republic of Korea). Briefly, a mixture of the twelve dried components (100 g; weight ratios shown in Table 1) was extracted in 800 ml of 30% ethanol by heating at 80°C for 4 h, and extracts were filtered twice through a filter paper (40 *μ*m, Whatman, Buckinghamshire, UK). Filtrates were concentrated under reduced pressure using a rotary vacuum evaporator (EYELA, Tokyo, Japan) at 40~45°C, and the concentrate was lyophilized using a freeze dryer (EYELA, Tokyo, Japan). The yield of the GST extract (dried powder) was 22.7% of the weight of the dried starting materials.

### 2.4. Cell Culture

SH-SY5Y cells, a human neuroblastoma cell line, were purchased from the Korean Cell Line Bank (Seoul, Republic of Korea). Cells were cultured in Dulbecco's modified Eagle's medium (DMEM, Welgene, Gyeongsan, Republic of Korea), supplemented with 10% fetal bovine serum (FBS, Thermo Scientific, Waltham, USA) and 1% penicillin/streptomycin. Cultures were maintained at 37°C in a CO_2_ incubator with a controlled humidified atmosphere composed of 95% air and 5% CO_2_. FBS, cell culture media, penicillin/streptomycin, and all other reagents used for cell culture studies were purchased from HyClone Laboratories (Logan, USA).

### 2.5. Cell Viability Assay

Cell viabilities were evaluated using a 3-(4,5-dimethylthiazol-2-yl)-2,5-diphenyltetrazolium bromide (MTT, Sigma-Aldrich, St. Louis, USA) assay. SH-SY5Y cells were plated at a density of 3-5 × 10^3^ cells/well and pretreated with either dimethyl sulfoxide (DMSO, Junsei Chemical Co., Tokyo, Japan) or GST (10-300 *μ*g/ml) for 1 h and then incubated in the presence or absence of 1 mM MPP^+^ (Sigma-Aldrich, St. Louis, USA) for 24 h. Viable cells were then stained with MTT solution (0.2 mg/ml, 3 h), and formazan crystals were dissolved in 100 *μ*l of 0.1% DMSO. Absorbance was measured at 540 nm using a multimode microplate reader (Tecan, Morrisville, USA).

### 2.6. Measurement of Mitochondrial Membrane Potentials

Mitochondrial membrane potentials were measured by flow cytometry using rhodamine 123 (Sigma-Aldrich, St. Louis, USA), a membrane-permeable cationic fluorescent dye. SH-SY5Y cells were stained with 0.05 *μ*g/ml of rhodamine 123 for 1 h and harvested by trypsinization. After washing with phosphate-buffered saline (PBS) containing 1% FBS, changes in mitochondrial membrane potentials were examined by measuring fluorescence intensities using a CytoFLEX flow cytometer (Beckman Coulter Inc., Brea, USA).

### 2.7. Measurement of ROS Production

SH-SY5Y cells were plated at a density of 2 × 10^5^ cells/well for 24 h. Cells were incubated with 100 *μ*g/ml of GST for 1 h and later treated with 1 mM MPP^+^ for 18 h. After the treatment, cells were stained with 5 *μ*M of 2′, 7′-dichlorodihydrofluorescein diacetate (H_2_DCF-DA, Sigma-Aldrich, St. Louis, USA) for 30 min at 37°C. For each analysis, a total of 10,000 events were recorded. Fluorescence intensity in the cells was measured using a CytoFLEX flow cytometer.

### 2.8. Western Blot Analysis

Samples were lysed with RIPA buffer (Thermo Scientific, Waltham, USA) containing phosphatase and protease inhibitor cocktail (Gen-DEPOT, Katy, USA), and then protein concentrations were assessed using a BCA protein assay kit (Thermo Scientific, Waltham, USA). Equal amount of proteins were separated by 10% SDS-PAGE, transferred onto PVDF membranes (Millipore, Burlington, USA), and blocked with 5% skim milk. Membranes were incubated with primary antibodies overnight at 4°C, washed 3 times with PBS containing 0.1% Tween 20, and incubated with HRP-conjugated secondary antibodies (Novus, Littleton, USA) for 1 h at room temperature. Detection was performed using an enhanced chemiluminescence system (Amersham Biosciences, Little Chalfont, UK). The primary antibodies used in this study were as follows: *β*-actin was purchased from Santa Cruz (Santa Cruz, USA); TH was obtained from Novus (Littleton, USA); procaspase-3, Bcl-2, phospho-Akt, Akt, phospho-ERK1/2, and ERK1/2 were obtained from Cell Signaling Technologies (Danvers, USA). The band intensity of the detected proteins were measured by Image J version 1.8.

### 2.9. Statistical Analysis

All the data are shown as means ± SEM. One-way ANOVA followed by Bonferroni's multiple comparison tests was performed to determine the significance of differences using GraphPad Prism software (GraphPad Software Inc., La Jolla, USA). Statistical significance was set at* p* < 0.05 for all analyses.

## 3. Result

### 3.1. GST Improves Motor Function Impairment in Parkinsonian Mice

We performed the pole test and rotarod test to examine the effect of GST on improvement of motor function. First of all, mice in the MPTP group spent 50% less time on the rotarod compared to the mice in the control group. This shows that MPTP resulted in motor dysfunction in mice consequently (*p* < 0.01, Figure 1(b)). Compared to MPTP group, motor function of mice was recovered to normal level in MPTP+GST group (*p *< 0.05, Figure 1(b)). Similarly, pole test showed that administration of GST reduced total time spent until reaching the floor compared with mice in MPTP group, which means that GST treatment ameliorated motor complications caused by MPTP (*p* < 0.001 control versus MPTP and* p* < 0.01 MPTP versus MPTP+GST, Figure 1(c)). Next, we performed western blot analysis for all the experimental groups of mice. Compared with the control group, the level of TH was about 60% lower in the MPTP group. Importantly, we could find increased level of TH in the GST administered MPTP group compared with the MPTP group (*p* < 0.01 Control versus MPTP and* p* < 0.05 MPTP versus MPTP+GST, Figure 1(d)).

### 3.2. Effects of GST on MPP^+^-Induced Cell Death in SH-SY5Y Cells

**Figure 2:**
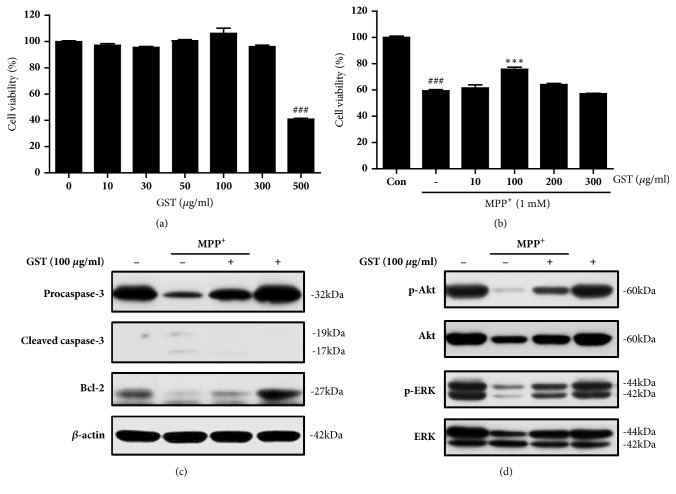
**Effects of GST extract on MPP** ^+^ **-induced cell death in SH-SY5Y cells. **(a) Effects of GST extract on SH-SY5Y cell viability. Cells were treated with various concentrations of GST extract (10, 30, 50, 100, 300, or 500 *μ*g/ml) for 24 h. Cell viability was determined by MTT assay. Values were expressed as percentages of the control. (b) Effects of GST extract against MPP^+^-induced neurotoxicity in SH-SY5Y cells. SH-SY5Y cells were pretreated with GST (10, 100, 200, or 300 *μ*g/ml) for 1 h and then exposed to 1 mM MPP^+^ for 24 h. The cell viability was measured using an MTT assay. ###* p* < 0.001, compared to control group; ∗∗∗* p* < 0.001, compared to MPP^+^ treatment, analyzed by one-way ANOVA, followed by Bonferroni's* post hoc* test. (c) Effects of GST on MPP^+^-induced apoptosis in SH-SY5Y cells. The expression of apoptosis-related proteins were measured by western blot in cells treated with 100 *μ*g/ml GST in the absence or presence of 1 mM MPP^+^ for 24 h. (d) Effects of GST on MPP^+^-induced changes of cell signaling proteins in SH-SY5Y cells. The expression of cell proliferation-related proteins was assayed by western blot in cells treated with 100 *μ*g/ml GST in the absence or presence of 1 mM MPP^+^ for 24 h.

To evaluate cytotoxicity of GST, SH-SY5Y cells were treated with various concentrations of GST extract (10, 30, 50, 100, 300, or 500 *μ*g/ml) for 24 h and then the cell viability was measured using MTT assay. GST concentration ranged from 10 to 300 *μ*g/ml and did not affect viability of SH-SY5Y cells, whereas 500 *μ*g/ml of GST treatment reduced the cell viability (*p* < 0.001, Figure 2(a)). Next, to examine the effect of GST against MPP^+^-induced neurotoxicity, SH-SY5Y cells were treated with GST (10, 100, 200, or 300 *μ*g/ml) for 1 h and then further exposed to 1 mM MPP^+^ for 24 h. As a result, 100 *μ*g/ml of GST pretreatment showed significant increase in cell viability against MPP^+^-induced cell death. According to these results, 100 *μ*g/ml of GST was selected as an effective dose for further experiments (*p* < 0.001, Figure 2(b)). And, the effect of GST on apoptosis induced by MPP^+^ was investigated by western blot analysis of apoptosis-related proteins expression. As a result, MPP^+^ reduced expression level of procaspase-3 and Bcl-2, whereas cleaved caspase-3 was increased (Figure 2(c)). And they were altered by GST pretreatment (Figure 2(c)). To test cell signaling proteins related to cell proliferation, we examined Akt and ERK phosphorylation levels by western blot analysis. In the result, both phosphorylation levels of Akt and ERK were decreased by MPP^+^, and these were altered by GST pretreatment (Figure 2(d)). Overall, these results suggest that GST recovered cell viability against MPP^+^-toxicity possibly via change of apoptosis- and cell proliferation-related proteins expression level.

### 3.3. Effects of GST on MPP^+^-Induced Oxidative Stress in SH-SY5Y Cells

**Figure 3:**
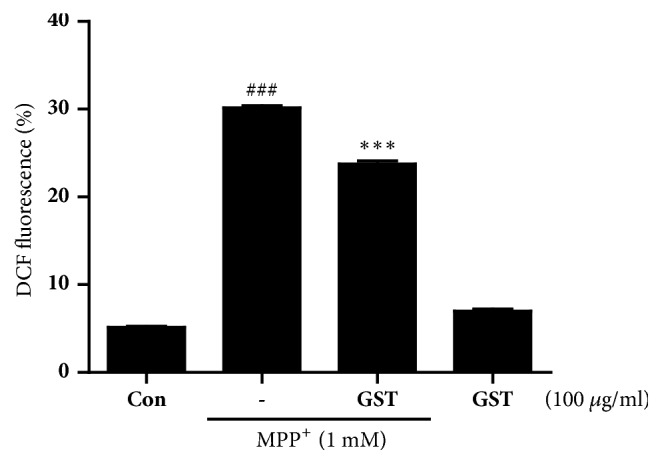
**Effects of GST extract on MPP** ^+^ **-induced ROS generation in SH-SY5Y cells.** Cells were pretreated with 100 *μ*g/ml GST for 1 h and then stimulated with or without 1 mM MPP^+^ for 18 h. After staining with 5 *μ*M H_2_DCF-DA, ROS production was monitored by measuring intensity of DCF fluorescence in cells using a flow cytometer. ###* p* < 0.001, compared to control group; ∗∗∗* p* < 0.001, compared to MPP^+^ treatment, analyzed by one-way ANOVA, followed by Bonferroni's* post hoc* test.

To further investigate the protective effects of GST against oxidative stress, intracellular reactive oxygen species (ROS) levels were assessed by the flow cytometric assay using H_2_DCF-DA. SH-SY5Y cells were pretreated with 100 *μ*g/ml of GST for 1 h and then stimulated with or without 1 mM MPP^+^ for 18 h. After staining with 5 *μ*M H_2_DCF-DA, ROS production was monitored by measuring intensity of DCF fluorescence in cells using the flow cytometer. The results showed that 1 mM MPP^+^ treatment induced about 30% ROS generation (*p* < 0.001 Control versus MPP^+^), whereas pretreatment of GST significantly inhibited MPP^+^-induced ROS increment (*p* < 0.001 MPP^+^ versus MPP^+^+GST, Figure 3).

### 3.4. Effects of GST on MPP^+^-Induced Mitochondrial Dysfunction in SH-SY5Y Cells

**Figure 4:**
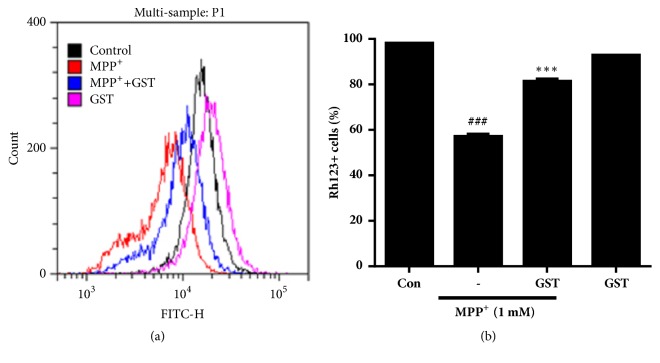
**Effects of GST extract on MPP** ^+^ **-induced mitochondrial dysfunction in SH-SY5Y cells.** Cells were pretreated with 100 *μ*g/ml GST for 1 h and then stimulated with or without 1 mM MPP^+^ for 18 h. After staining with rhodamine 123, cells were subjected on flow cytometer. ###* p* < 0.001, compared to control group; ∗∗∗* p* < 0.001, compared to MPP^+^ treatment, analyzed by one-way ANOVA, followed by Bonferroni's* post hoc* test.

To examine whether GST has protective effect on mitochondrial dysfunction by MPP^+^ in SH-SY5Y cells, mitochondrial membrane potentials were measured using the flow cytometry. SH-SY5Y cells were treated with 100 *μ*g/ml of GST for 1 h and then stimulated with or without 1 mM MPP^+^ for 18 h. After staining with rhodamine 123, cells were loaded on the flow cytometer. MPP^+^ treatment increased the proportion of cells with low rhodamine 123 fluorescence intensity (43.1 ± 0.4%,* p* < 0.001 control versus MPP^+^), as indicated by mitochondrial damage and dysfunction (Figure 4). In contrast, GST pretreatment significantly reduced the proportion of cells with low Rh123 fluorescence intensity (18.5 ± 0.9%,* p* < 0.001 MPP^+^ versus MPP^+^+GST). These results show that GST alleviated MPP^+^-induced mitochondrial dysfunction.

### 3.5. Effects of GST on the Akt and ERK Signaling in SH-SY5Y Cells

**Figure 5:**
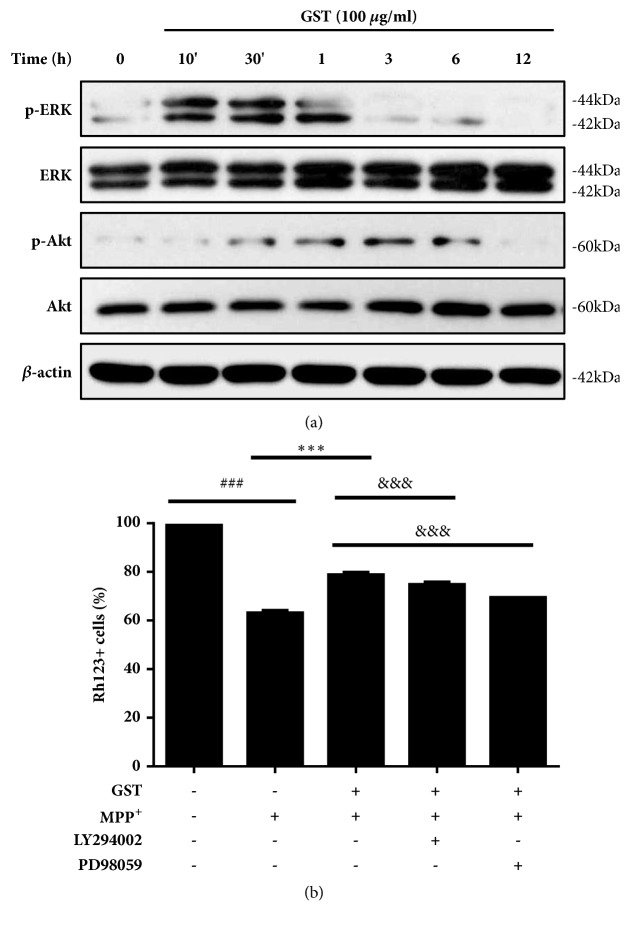
**Effects of GST on the Akt and ERK signaling in SH-SY5Y cells.** (a) Akt and ERK activation by GST. SH-SY5Y cells were treated with 100 *μ*g/ml GST for the indicated times. The protein levels of Akt and ERK and their phosphorylated forms were analyzed by western blot. (b) Role of Akt and ERK activation by GST in mitochondrial function. After LY294002 and PD98059 pretreatment (10 *μ*M, 1 h), cells were incubated with GST for 1 h and then treated with 1 mM MPP^+^ for 18 h. After staining with rhodamine 123, cells were subjected on flow cytometer. ###* p* < 0.001 compared to control group, ∗∗∗* p* < 0.001 compared to MPP^+^ treatment, &&&* p* < 0.001 compared to MPP^+^+GST treatment, analyzed by one-way ANOVA, followed by Bonferroni's* post hoc* test.

To elucidate the cellular mechanism linked with the cytoprotective effects of GST, we examined cell survival-related signaling pathways by western blot analysis. According to the result, we found that expressions of phosphorylated-Akt and -ERK were significantly increased by treatment of 100 *μ*g/ml of GST (Figure 5(a)). The phosphorylation of ERK was strongly expressed in 10 min after GST treatment and sustained for 3 h. Meanwhile, Akt phosphorylation started 30 min after GST treatment, peaked at 3 h, and lasted for 6 h.

To investigate the role of ERK and Akt activation in the cytoprotective effect of GST, mitochondrial membrane potentials were measured after treating SH-SY5Y cells with specific inhibitors of each protein. As shown in Figure 5(b), the protective effect of GST against the toxicity of MPP^+^ was partially blocked by PD98059 and LY294002, selective inhibitors of ERK and Akt, respectively. These observations suggest that the neuroprotective effects of GST extract were associated at least in part with ERK and Akt activation.

## 4. Discussion

It has been thought that loss of dopaminergic neurons in the substantia nigra, as a result of oxidative stress, is one of the potential causes of PD [12]. For the treatment of PD, a lot of drugs, including dopaminergic drugs and nondopaminergic drugs, have been widely used in last few decades; however, detrimental motor complications of the current PD treatment medications are still considerable [13]. There are a number of on-going researches aiming for discovering alternative PD treatments with fewer side effects. Herbal medicines are one of the alternative treatments of PD and many other diseases.

GST is modified formula of SDT with two additional herbs,* Uncaria rhynchophylla *and* Gastrodia elata* Blume. SDT has been used for patients diagnosed with deficiency of qi and blood in East Asian medicine, and* Uncaria rhynchophylla* and* Gastrodia elata* Blume have been known to control tremor in the traditional herbal medicine [5, 8, 9, 14]. Because patients diagnosed with PD are generally considered to exhibit unbalanced qi and blood with tremor in oriental medicine, GST has been used in order to alter the symptoms of PD under clinical conditions.

In the present study, we examined the effect of GST* in vivo* and* in vitro* by using MPTP-induced PD mice model and MPP^+^-induced cell death in SH-SY5Y cells. In the animal study, improvement in motor function of PD mice model was evident in rotarod test and pole test after GST treatment. Neuroprotective effect of GST was suggested by the* in vitro* study. MTT assay results showed that GST increased viability of cells and inhibited apoptosis against MPP^+^ toxicity. Also, decrease in proportion of low fluorescence intensity was detected after GST pretreatment, indicating that mitochondrial dysfunction was attenuated by GST pretreatment. MPP^+^ toxicity caused increment of ROS and resulted in oxidative stress in SH-SY5Y cells. By using H_2_DCF-DA staining, it was shown that GST inhibited MPP^+^-induced increase of ROS.

We could also see that protective effect of GST was attenuated when selective inhibitors antagonized ERK or Akt. Akt and ERK are mitogen activated protein kinases, which are known to regulate cell proliferation and cell differentiation by activating downstream CRE gene pathway. Akt- and ERK-initiated transcription of CRE-binding protein is known to induce upregulation of Bcl-2 which is antiapoptotic and prosurvival [15, 16]. Upregulation of Bcl-2 controls mitochondrial dysfunction by regulating activation of BAX and BAK, which are mitochondrial membrane proteins [17]. Thus, our results possibly indicate that GST recovered mitochondrial dysfunctions via Akt and ERK signaling pathways.

Still the main component of GST which shows these effects on PD remains unclear, but previous studies have drawn possible candidates. One candidate chemical of Rehmannia, catalpol, is an ingredient of SDT. Catalpol is known to have neuroprotective effect in neurotoxicity by attenuating mitochondrial dysfunction and MAO-B activity [18]. Furthermore, in Alzheimer's disease mouse model, SDT attenuates *β*-amyloid deposits [19] and catalpol has shown its neuroprotective effect from *β*-amyloid induced neuron injury and following cognitive functions in aged rats [20]. Further researches are needed to find a main component of herbal formula for development of optimal drug for PD.

## 5. Conclusion

We examined membrane potential, ROS, apoptosis- and cell proliferation-related proteins in order to see the effect of GST on PD by using both animal model and* in vitro* method. As a result, it was revealed that specific dose of GST increased cell viability and ameliorated MPTP-induced motor dysfunction. MPP^+^-induced mitochondrial dysfunction was prevented in SH-SY5Y cells and dopaminergic neurons were protected in PD mouse model. Our data suggests that GST may be a potential candidate for alternative treatment of PD.

## Figures and Tables

**Table 1:** The compositions of GST.

**Latin Name**	**Scientific name (Family name)**	**Ratio**
Ginseng Radix Alba	*Panax ginseng* C. A. Meyer (Araliaceae)	3
Atractylodis Rhizoma Alba	*Atractylodes macrocephala* Koidzumi (Compositae)	2
Hoelen	*Poria cocos *Wolf (Polyporaceae)	2
Glycyrrhizae Radix et Rhizoma	*Glycyrrhiza uralensis* Fischer (Leguminosae)	2
Rehmanniae Radix Preparata	*Rehmannia glutinosa *Liboschitz ex Steudel (Scrophulariaceae)	2
Angelicae Gigantis Radix	*Angelica gigas* Nakai (Umbelliferae)	2
Cnidii Rhizoma	*Cnidium officinale* Makino (Umbelliferae)	2
Paeoniae Radix	*Paeonia lactiflora *Pallas (Paeoniaceae)	2
Astragali Radix	*Astragalus membranaceus *Bunge (Leguminosae)	3
Bupleuri Radix	*Bupleurum falcatum* Linne (Umbelliferae)	2
Uncariae Ramulus et Uncus	*Uncaria rhynchophylla* (Rubiaceae)	2
Gastrodiae Rhizoma	*Gastrodia elata *Blume (Orchidaceae)	2

## Data Availability

All the data used to support the findings of this study are included within the article.
